# Ice-cream used as cryotherapy during high-dose melphalan conditioning reduces oral mucositis after autologous hematopoietic stem cell transplantation

**DOI:** 10.1038/s41598-021-02002-x

**Published:** 2021-11-18

**Authors:** Marcin Jasiński, Martyna Maciejewska, Anna Brodziak, Michał Górka, Kamila Skwierawska, Wiesław W. Jędrzejczak, Agnieszka Tomaszewska, Grzegorz W. Basak, Emilian Snarski

**Affiliations:** 1 Department of Hematology, Transplantation and Internal Medicine, Medical University of Warsaw, Warsaw, Poland; 2 Department of Experimental and Clinical Physiology, Laboratory of Centre for Preclinical Research, Medical University of Warsaw, Warsaw, Poland

**Keywords:** Cancer, Haematological cancer

## Abstract

Oral mucositis (OM) is one of the most frequent adverse events of high-dose conditioning chemotherapy with melphalan prior to autologous hematopoietic stem cell transplantation (AHSCT). It significantly reduces the patients’ quality of life. One of the preventive strategies for OM is cryotherapy. We retrospectively analyzed whether commercially available ice-cream could prevent OM during the melphalan infusion. We retrospectively analyzed 74 patients after AHSCT to see whether there is any correlation between OM and cryotherapy (ice-cream), melphalan dose (140 mg/m^2^ or 200 mg/m^2^). The incidence of OM in our study inversely correlated with cryotherapy in the form of ice-cream. Out of 74 patients receiving conditioning chemotherapy with high-dose melphalan, 52 received cryotherapy. Fifteen patients in the cryotherapy group (28.84%) developed OM, whereas 13 patients (59.09%) developed it in the group without cryotherapy. In a multiple linear regression test cryotherapy remained a significant protective factor against OM (*p* = 0.02) We have also seen the relationship between melphalan dose with OM (*p* < 0.005). Cryotherapy in the form of ice-cream is associated with a lower rate of OM and, therefore, could potentially be used as a cost-effective, less burdensome, and easy to implement method in prevention of oral mucositis.

## Introduction

Autologous hematopoietic stem cell transplantation (AHSCT) is part of standard therapy in patients with multiple myeloma, amyloidosis, systemic sclerosis or POEMS syndrome^1,2^. Due to the use of high-dose of conditioning chemotherapy regimens, patients experience a wide variety of toxicities. Oral mucositis (OM) is one of the most common side effects of this treatment, and is reported by patients as the most deteriorating and decreasing the quality of life^3^. The OM often causes the need for parenteral nutrition but also increases the risk of bacterial translocation and sepsis during cytopenia^4^. In the most severe cases it may require parenteral opioids to relieve the patients’ pain what is associated with the risk of adverse events^5,6^. One of the strategies used to prevent OM is cryoprotection^7^. Typical cryoprotection protocols entail prolonged use of ice chips or ice cubes, which are not well received by patients^8^. While this approach is effective^9^, it is still not widely applied in transplantation centers. The mechanism of ice chips' action is vasoconstriction, which stops the inflammation in the region of the oral cavity mucosa and reduces the contact of toxic drugs with the mucous layer^10^. The AHSCT in multiple myeloma might be a spot-on indication for cryotherapy as melphalan used as a standard conditioning regimen has a short half time and thus, the maximal toxicity is limited to the few hours after the start of infusion. In fact, it has been proven that cryotherapy during high-dose melphalan administration effectively reduces incidence and severity of OM in patients undergoing AHSCT^8,11^. However due to long time of melphalan infusion, some patients complain of a cold sensation and stop the use of ice chips before infusion ends^8^.

Another, more patient-friendly and easy to comply approach to cryotherapy deployed by some centers is the use of ice cream. This is especially common in pediatric centers. Surprisingly, we could not identify in literature searches any references that could provide evidence that this procedure lowers the chances for oral mucositis in adult patients receiving chemotherapy.

In our work, we analyzed the impact of consuming commercially available ice-cream during the melphalan infusion on mucositis after the AHSCT.

## Methods

We retrospectively analyzed the medical data of the patients who underwent AHSCT with non-cryopreserved hematopoietic stem cells in our clinic between November 2017 and December 2020. The inclusion criterium was the availability of the data on the cryotherapy in the medical records and 74 out of all 87 patients met the inclusion criteria. Sixty-three patients had multiple myeloma (MM), 4 patients had amyloidosis with MM (AL + MM), 3 patients had amyloidosis (AL), 3 patients had systemic sclerosis (SSc) and one patient had POEMS (Polyneuropathy, Organomegaly, Endocrinopathy, Myeloma protein, Skin changes) syndrome. As conditioning therapy, the patients received 200 mg/m^2^ or 140 mg/m^2^ melphalan, depending on the clinical picture of the disease and comorbidities. The dose was adjusted in accordance with EBMT CALM study results^12^. Patients without any of OM symptoms were classified as 0, while patients with the most severe, life-threatening OM were assessed as IV in CTC-AE v 5.0 scale. Oral mucositis assessment was done every day during the morning doctor’s round by attending physician. The patient's highest recorded score was documented in the discharge letter and used for the analysis in this study. All methods were carried out in accordance with relevant guidelines and regulations. All patients provided informed consent for the treatment and for the use of medical data for scientific purposes.

### Cryotherapy with ice-cream

The patients received cryotherapy with ice-cream on demand or as requested by the physician for the prevention of oral mucositis. The ice-cream was given from the beginning of the melphalan infusion. The protocol consisted of 3 ice-cream doses chosen by the patient from ice-cream commercially available at the hospital cafeteria. Patients received ice-cream in the form of popsicles and dairy-containing products as well. The consecutive ice-creams were given on patient demand. Patients were asked to eat slowly, thawing the ice-cream in the mouth. The compliance with the protocol was not measured. For the duration of neutropenia all patients received the same oral care which comprised of octenidine and calcium phosphate rinses.

### Statistical analysis

To test whether cryotherapy remained a significant protective factor against OM after taking into account melphalan dose we used a multiple linear regression test. The comparison of age between groups with or without OM was made with the two-tailed Mann–Whitney U Test (Calculator: https://www.socscistatistics.com/tests/mannwhitney/).

### Ethics approval

This research study was conducted retrospectively from data obtained for clinical purposes. Ethical approval was waived by the local Ethics Committee of the Medical University of Warsaw in view of the retrospective nature of the study and all the procedures being performed were part of the routine care.

## Results

**Figure 1:**
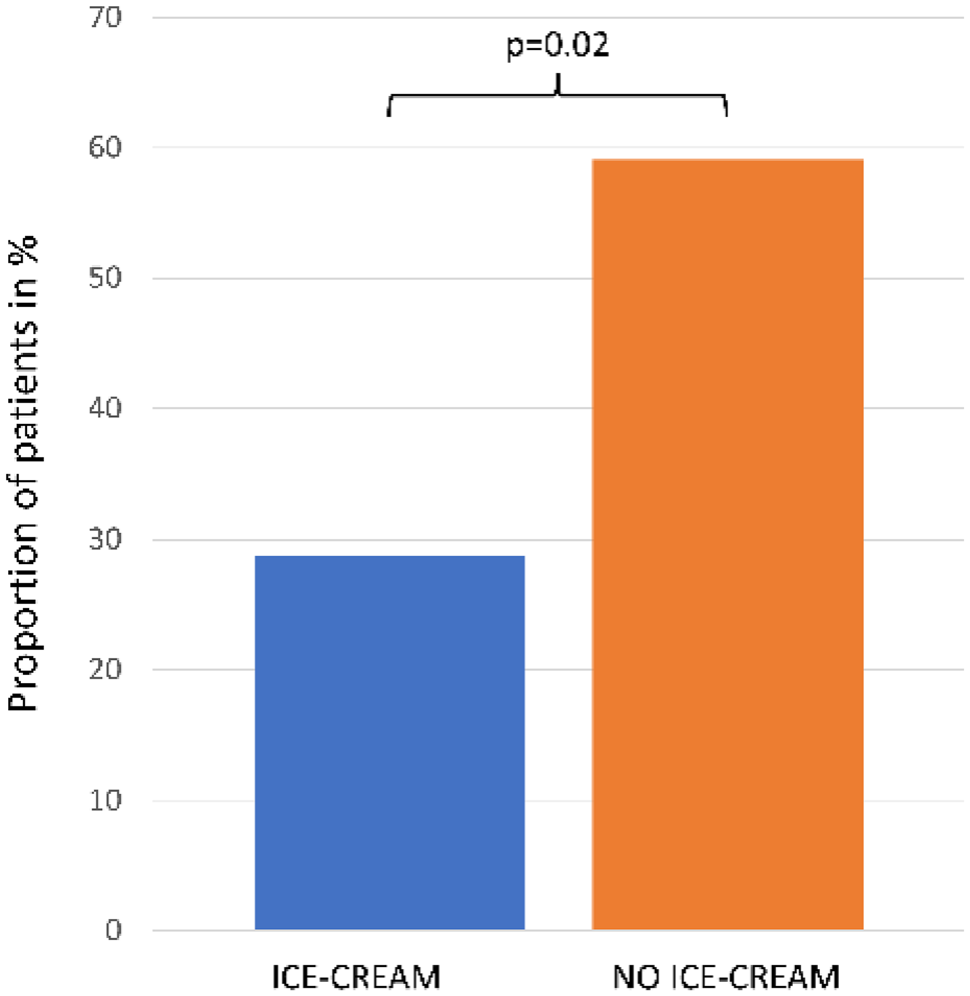
Proportion of patients with oral mucositis on and without ice-cream cryotherapy. 28.85% patients who received ice-cream cryotherapy developed OM, whereas 59.09% developed it in the group with no cryotherapy. In a multiple linear regression test cryotherapy remained a significant protective factor against OM (*p* = 0.02).

The mean age of patients was 58.1 years (Table 1). Overall, 28 out of 74 patients (37.84%) developed OM after AHSCT. Fifty-two patients received cryotherapy during infusion of melphalan, whereas 22 of them did not. In the group with cryotherapy, 15 of 52 (28.85%) patients developed OM, while in the group without cryotherapy, 13 of 22 (59.09%). In a multiple linear regression test cryotherapy remained a significant protective factor against OM (*p* = 0.02) (Fig. 1). No mucosal-injury-related sepsis was observed in the study population. The median length of hospitalization in patients without OM was 17 days whereas in patients with OM—19 days (whole population—18 days). In the studied population only 3 patients (4.1%) required total parenteral nutrition (TPN). Two of them received TPN because of the grade 3 and 4 OM in the CTC-AE v 5.0. scale and were fed parenterally for 21 and 12 days respectively. The third patient was given with TPN for 11 days because of subileus. It is also worth mentioning that in the control group 3 patients were classified as G3 and 1 patient was classified as G4 OM, whereas in the cryotherapy group 2 and 0 patients respectively.

**Table 1:** Baseline characteristics of analysed patients with or without cryotherapy.

Variable	Group	Cryotherapy N = 52	No cryotherapy N = 22
Age in years	Mean	57.8	58.7
	Range	26–70	37–68
Indication for AHSCT n (%)	MM	44 (84.62)	19 (86.36)
	AL + MM	3 (5.77)	1 (4.55)
	AL	2 (3.85)	1 (4.55)
	SSc	3 (5.77)	0 (0)
	POEMS	0 (0)	1 (4.55)
Melphalan dose n (%)	140/m^2^	33 (63.46)	11 (50)
	200/m^2^	19 (36.54)	11 (50)
Grades of OM n (%)	G0	37 (71.15)	9 (40.91)
	G1	11 (21.15)	4 (18.18)
	G2	2 (3.85)	5 (22.73)
	G3	2 (3.85)	3 (13.64)
	G4	0 (0)	1 (4.55)

*OM* oral mucositis, *AHSCT* autologous hematopoietic stem cell transplantation, *MM* multiple myeloma, *AL* light chain amyloidosis, *SSc* systemic sclerosis, *POEMS* polyneuropathy, organomegaly, endocrinopathy, monoclonal gammopathy, skin abnormalities.

## Discussion

The study provides evidence that cryotherapy can prevent OM in patients undergoing AHSCT. In our analysis, we have demonstrated that the use of commercially available ice-cream in patients receiving high-dose chemotherapy conditioning regimens significantly reduces the risk of OM. The protocol used in our study is simple, easy to implement, cost-efficient, and less burdensome for the patients. Our findings are in line with the data on cryotherapy effectiveness as OM prophylaxis. A recent systematic review on cryotherapy in patients undergoing AHSCT using high-dose melphalan conditioning regimen upgraded this strategy's level of evidence to *recommendatio*n^13^. A meta-analysis of randomised controlled trials from 2015 comparing the effect of oral cryotherapy with no treatment in ASCT patients confirmed cryotherapy effectiveness in reducing incidence of severe OM, duration of use of parenteral nutrition and hospitalization length^14^. However, the aforementioned studies concentrated on cryotherapy protocols that included the use of ice chips. Therefore, our analysis adds additional data on the effectiveness of cryotherapy protocol with the use of commercially available ice cream. Considering the lack of studies on this type of cryotherapy strategy, our study fills a knowledge gap in this area. The relatively small groups and retrospective methodology are the most important limitations of this study. The results should be validated in a prospective randomized study comparing the effectiveness of different cryotherapy protocols to confirm non-inferiority of the strategy based on the use of commercially available ice-cream. The influencing factor was not surprisingly higher mg/kg melphalan dose, which stays in line with previous findings^15^.

In summary, we have shown that the application of commercially available ice-cream during short melphalan infusions can contribute to a lower rate and severity of mucositis in patients undergoing autologous HSCT.

## Data Availability

Data from the study can be obtained upon request by the corresponding author.
